# Protease circuits for processing biological information

**DOI:** 10.1038/s41467-020-18840-8

**Published:** 2020-10-06

**Authors:** Brandon Alexander Holt, Gabriel A. Kwong

**Affiliations:** 1 Wallace H. Coulter Department of Biomedical Engineering, Georgia Tech College of Engineering and Emory School of Medicine, Atlanta, GA 30332 USA; 2 Parker H. Petit Institute of Bioengineering and Bioscience, Atlanta, GA 30332 USA; 3 Institute for Electronics and Nanotechnology, Georgia Tech, Atlanta, GA 30332 USA; 4 Integrated Cancer Research Center, Georgia Tech, Atlanta, GA 30332 USA; 5 The Georgia Immunoengineering Consortium, Emory University and Georgia Tech, Atlanta, GA 30332 USA

**Keywords:** Biomaterials, Synthetic biology

## Abstract

Engineered biocircuits designed with biological components have the capacity to expand and augment living functions. Here we demonstrate that proteases can be integrated into digital or analog biocircuits to process biological information. We first construct peptide-caged liposomes that treat protease activity as two-valued (i.e., signal is 0 or 1) operations to construct the biological equivalent of Boolean logic gates, comparators and analog-to-digital converters. We use these modules to assemble a cell-free biocircuit that can combine with bacteria-containing blood, quantify bacteria burden, and then calculate and unlock a selective drug dose. By contrast, we treat protease activity as multi-valued (i.e., signal is between 0 and 1) by controlling the degree to which a pool of enzymes is shared between two target substrates. We perform operations on these analog values by manipulating substrate concentrations and combine these operations to solve the mathematical problem Learning Parity with Noise (LPN). These results show that protease activity can be used to process biological information by binary Boolean logic, or as multi-valued analog signals under conditions where substrate resources are shared.

## Abstract (web summary)

In contrast to genetic circuits, here the authors develop protein biocircuits based on proteases. They show these activity-based circuits can execute Boolean logic for programmable drug delivery and perform fuzzy logic to solve a mathematical oracle problem, Learning Parity with Noise.

## Introduction

The forward engineering of cellular^1–4^ and molecular^5–10^ computing systems is driven by integrating elementary biological parts to produce high-level functions^11,12^. The development of foundational components, such as molecular logic gates^9^ and genetic clocks^13,14^, have enabled the design of biocircuits with increasing complexity, including the ability to solve mathematical problems^15^, build autonomous robots^16^, and play interactive games^17^. To date, the majority of biocircuits are implemented in platforms that operate on the genetic circuit analogy^18^, which require genome or protein engineering^19–22^. These genetic circuits process biological signals (e.g., pH, temperature, chemical concentrations, etc.) by receiving information via promoters that induce gene expression, and output information by expressing reporters (e.g., GFP) or effector molecules (e.g., therapeutics)^19^. Traditionally, genetic circuits digitize these molecular signals as two-valued states (e.g., low vs. high concentration is represented as state 0 or 1, respectively) to allow operations to be carried out by Boolean logic (e.g., AND gates)^1,23,24^.

By contrast, analog circuits are designed to represent variables using the entire range of continuous values (i.e., between 0 and 1) rather than two-valued integers (i.e., either 0 or 1)^11,24,25^. Analog circuits are better suited for processing problems with uncertainty by implementing so-called “fuzzy logic”, which assigns weights to each possible value that a variable can hold^26^. Several molecular analog circuits have been implemented, including genetic circuits that carry out mathematical functions^27,28^ and DNA strand-displacement cascades to emulate neural networks^29^. Enzyme activity has also been used in analog biocircuits^10,30^ by making use of promiscuity, which is an enzyme’s capacity to recognize and catalyze different substrates^31–39^. Enzyme promiscuity bolsters evolutionary fitness and increases biological efficiency by using fewer enzymes to carry out the same number of reactions^39^. Importantly, promiscuity creates a shared resource environment where enzymes and substrates are in competition for binding partners depending on resource scarcity. For instance, when strong promoters are used in synthetic gene circuits, transcriptional and translational machinery are diverted to express the synthetic circuit, creating competition for RNA polymerases^40^ and ribosomes^41–43^. Consequently, the flow of biological information can be controlled by directing how resources are partitioned^44^. This control strategy is fundamental to the design of biological systems that implement analog operations such as stochastic biocircuits^45^, autonomous diagnostics^12^, and synthetic ribosomes that insulate genetic circuits^46^.

**Fig. 1:**
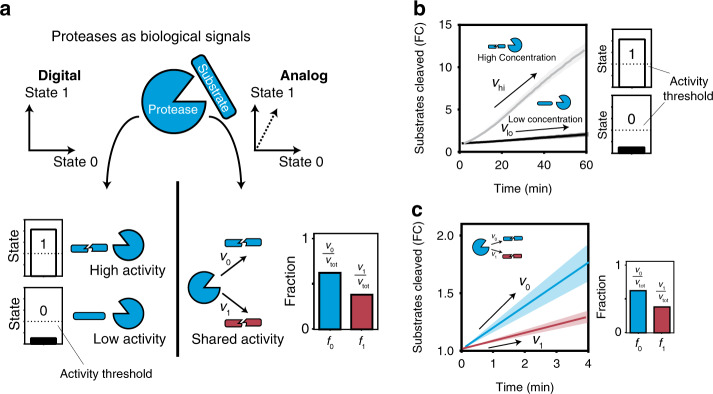
Protease activity as digital or analog signals. **a** (Left, top) A digital protease signal is exclusively two-valued (state 0 or state 1) (left, bottom) A digital protease signal in a state of either high or low protease activity, separated by an activity threshold (dotted line). (right, top) Analog signals are represented by a continuous value between two states (0 and 1) (dashed arrow). (right, bottom) An analog protease signal acting on two-state substrates has two cleavage velocities (*v*_0_ and *v*_1_), which represent the fraction of the protease pool cleaving either substrate (state-0 or 1). **b** Experimental data of a digital, two-valued protease signal. Protease (C1r) activity assay against substrate (LQRIYK), at high and low activity, which is controlled by the protease concentration. Activity threshold is used to separate state-1 (high activity) from state-0 (low activity). **c** Protease (Plasmin) activity assay against substrate state-0 (GLQRALEI) and state-1 (KYLGRSYKV). Relative velocities represent the fraction of the protease pool cutting in either state. *f*_0_ is the fraction of plasmin cleaving substrate 0 and *f*_1_ is the fraction of plasmin cleaving substrate 1. Data are presented as mean values ± standard deviation. Line shading in both panels represents standard deviation (*n* = 3 biologically independent samples). RFU stands for “Relative Fluorescence Unit”, which represents the amount (i.e., moles) of cleaved substrate and is plotted as fold change (FC) from initial fluorescence at time = 0. Source data are provided as a Source data file.

Building on these insights, we sought to construct biocircuits that use protease activity to process biological information under a digital or analog framework. We chose to use proteases because they are ubiquitous, comprise 2% of the human genome^47^, and have previously been used in genetic circuits to implement^48^ and control^49^ information-processing computations. Under a digital framework, we construct peptide-caged liposomes that treat protease activity as two-valued operations depending on the level of activity (i.e., 0 or 1 for low or high protease activity respectively) (Fig. 1a left, 1b). We show a biological application by integrating these peptide-caged liposomes into an analog-to-digital converter for autonomous drug delivery. By contrast, we demonstrate in a shared resource environment that the activity of a promiscuous protease is partitioned when more than one peptide substrate is present (Fig. 1a right, 1c). We show that the fraction of substrates being cleaved can be quantified as a continuous analog signal (i.e., value ranges from 0 to 1) depending on the relative substrate concentration. We use these analog operations to design a biological circuit to solve the mathematical problem learning parity with noise (LPN).

## Results

### A biological analog to digital converter

**Fig. 2:**
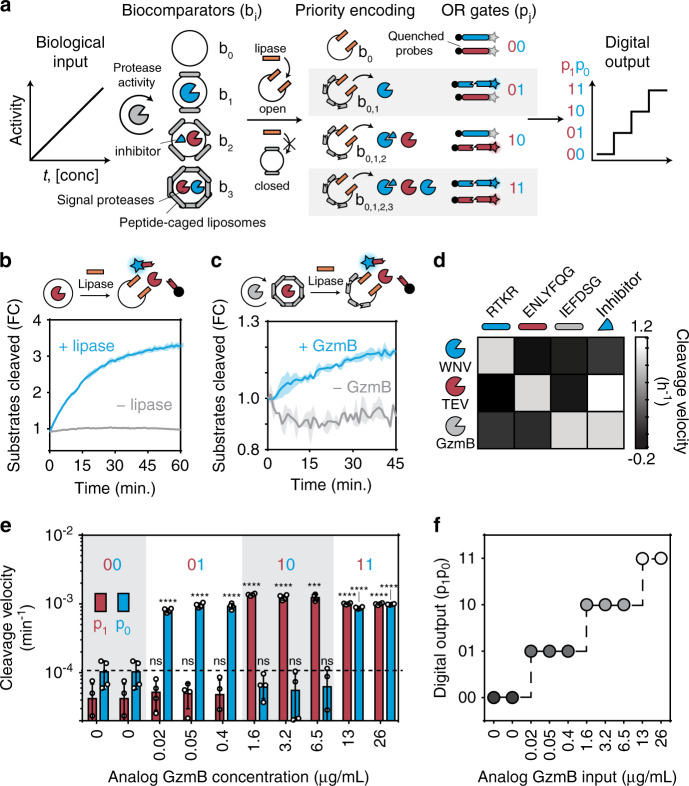
A biological ADC converts protease activity to a digital signal. **a** Biocircuit diagram depicting the conversion of a biological input (protease activity) into a digital output with biocomparators, priority encoding, and OR gates. Circular arrow around gray protease represents enzyme activity. **b** Bare or **c** peptide-caged liposomes opened by lipase (orange) or GzmB (gray) activity, respectively, release TEV protease (red) that cleaves a quenched peptide substrate. In **c**, liposomes (34 mM total lipid concentration) were loaded with TEV protease (1 μg protease/17 mmol lipids) and crosslinked with GzmB substrate peptide cage (peptide:liposome reaction ratio ~ 0.5 μmol/g). Fluorescent reporter is cleaved only in the presence of GzmB to degrade the peptide cage. Data is presented as mean (line) ± standard deviation (shading) from samples (*n* = 3 biologically independent samples) in **b**, **c**. **d** Protease orthogonality map measuring GzmB, WNV (blue), and TEV protease activity against respective substrates alone and in the presence of WNV protease inhibitor (blue triangle). **e** Increasing concentrations of GzmB across four orders of magnitude are input to the bioADC, and proteases p_0_ (WNV, blue) and p_1_ (TEV, red protease) are read out in a fluorescent assay using fluorescence-quenched substrates (RTKR and ENLYFQG). All data points are plotted as the slope of the best fit line to the cleavage velocities. Unpaired, one-way *t*-tests (*n* = 4 biologically independent samples) were performed between the condition with GzmB and the negative control (no GzmB) for each respective output (i.e., p_0_ or p_1_). Reported -values: from left to right starting with 0.02 µg/mL condition (blue bars): *p*-values = 9.21e-7, 3.27e-6, 1.33e-5, 0.111, 0.086, 0.130, 5.20e-7, and 3.35e-6; (red bars) *p*-values = 0.298, 0.326, 0.390, 1.150e-7, 5.870e-5, 0.000133, 1.690e-9, 7.040e-9. Biocomparator levels 0–3 are referenced by peptide cage reaction ratios, 0.0, 0.05, 5, and 5 × 10^2^ μmol/g (peptide:liposome), respectively. Dashed line represents activity threshold that separates an output of 1 (velocity > 10^−4^ min^−1^) from an output of 0 (velocity < 10^−4^ min^−1^). Bars are reported as mean ± standard deviation (error bars). **f** Digital, two-valued output as a function of input GzmB concentration. RFU stands for “Relative Fluorescence Unit”, which represents the amount (i.e., moles) of cleaved substrate and is plotted as fold change (FC) from initial fluorescence at time = 0. Velocity is calculated as the difference in signal over time, or quantity of substrates cleaved per unit time (i.e., [time]^−1^). n.s. not significant, *<0.05, **<0.01, ***<0.001, and ****<0.0001. Source data are provided as a Source data File.

A central function of complex circuits is the ability to store and manipulate digitized information; therefore, we first set out to construct a flash analog-to-digital converter (ADC) to convert biological signals into binary digits. An electronic ADC performs three major operations during signal conversion: voltage comparison, priority assignment, and digital encoding. An analog input voltage is first compared against a set of increasing reference voltages (*V*_0_ − *V*_i_) by individual comparators (*d*_0_ − *d*_i_) that allow current to pass if the input signal is greater than or equal to its reference value (Fig. S1). During priority assignment, only the activated comparator with the highest reference voltage, *d*_n_, remains on while all other activated comparators, *d*_*n*__ − 1_ − *d*_0_ are turned off. The prioritized signal is then fed into a digital encoder comprising OR gates to produce digital values. To design an ADC biocircuit using protease activity as the core signal, we constructed biological analogs of comparators by using liposomes locked by an outer peptide cage^50,51^ (Fig. 2a and Fig. S2a, b). With increasing peptide crosslinking densities, these biocomparators (*b*_0_ – *b*_i_) served to reference the level of input protease activity (GzmB) required to fully degrade the peptide cage (IEFDSGK, Table S1) and expose the lipid core (Fig. S2c), analogous to the reference voltages stored in electronic comparators. We used lipase^52^ as a Buffer gate to open all biocomparators with fully degraded cages (Fig. 2b, c and Fig. S3) and release a unique combination of inhibitors and signal proteases (WNV, TEV, and WNV inhibitor) that collectively act to assign priority to the highest activated biocomparator (*b*_n_) by inhibiting all signal proteases released from other biocomparators (*b*_0_ − *b*_*n* − 1_). To encode the prioritized signal into digital values, we designed a set of OR gates using orthogonal quenched substrates (RTKR and ENLYFQG) specific for the signal proteases (WNV and TEV, respectively, Fig. 2d) to provide fluorescent two-signal readouts (p_0_ − p_i_; Fig. S4a). Fully integrated, our biological ADC converted input protease levels (GzmB) across four orders of magnitude into digital outputs (Fig. 2e, f and Fig. S4b).

### An integrated bioADC to execute an antimicrobial program

**Fig. 3:**
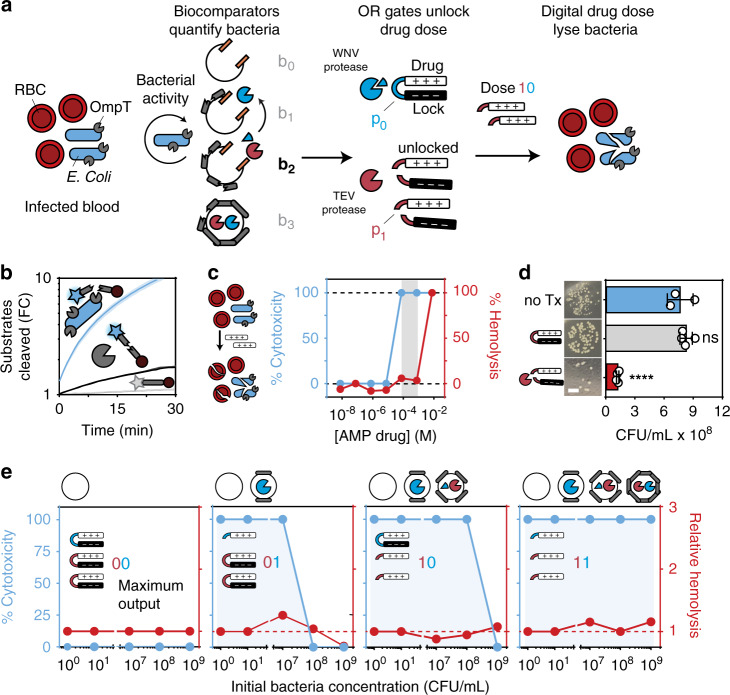
An integrated bioADC to execute an antimicrobial program. **a** Biocircuit depicting the use of an ADC to quantify bacteria and autonomously unlock digital drug doses. Circular arrow represents enzyme activity. **b** Cleavage assay measuring recombinant OmpT and live *E. coli* culture cleavage of substrate (RRSRRV) Data is presented as mean (line) ± standard deviation (shading) (*n* = 3 biologically independent samples). **c** EC50 measurement for drug cytotoxicity and hemolysis against *E. coli* and RBCs, respectively. Data is presented as mean ± standard deviation for cytotoxicity (blue) (for cytotoxicity *n* = 3 biologically independent samples, for hemolysis *n* = 2 biologically independent samples). Gray shading represents therapeutic window with 100% cytotoxicity and baseline hemolysis. **d** Viability of bacteria after treatment with locked drug and locked drug + protease (*n* = 4 biologically independent samples for treatment groups, gray, and red; *n* = 3 biologically independent samples for bacteria only, blue), one-way ANOVA and Dunnett’s multiple comparisons to bacteria only control; *p*-values reported for comparison between no Tx (blue) and with Tx (gray) is *p*-value =  0.4483, and between no Tx (blue) and with Tx + protease (red) is *p*-value = <0.0001. Scale bar = 4 mm. **e** Drug bacteria cytotoxicity and RBC hemolysis at five concentrations of bacteria with four different versions of the antimicrobial program each containing a different number of biocomparators. Data is presented as mean ± standard deviation for cytotoxicity (blue) (for cytotoxicity *n* = 3 biologically independent samples, for hemolysis *n* = 2 biologically independent samples). The four biocomparators, b_0_, b_1_, b_2_, and b_3_, were cross-linked at peptide cage reaction ratios, 0.0, 3.4 × 10^−2^, 3.4, and 340 μmol/g (peptide:liposome), respectively. RFU stands for “Relative Fluorescence Unit”, which represents the amount (i.e., moles) of cleaved substrate and is plotted as fold change (FC) from initial fluorescence at time = 0. n.s. not significant, *<0.05, **<0.01, ***<0.001, and ****<0.0001. Source data are provided as a Source data file.

To demonstrate a biological application, we next sought to interface our biological ADC with a living system for digital drug dosing. We rewired our ADC to autonomously quantify input bacterial activity and then output an anti-microbial drug dose to selectively clear red blood cells (RBCs) of bacteria (DH5α *Escherichia coli*) (Fig. 3a). To construct biocomparators with the ability to prioritize input levels of bacterial activity, we synthesized liposomes with peptide cages using a substrate (RRSRRVK) specific for the *E. Coli* surface protease OmpT^53,54^ (Fig. 3b). We synthesized a series of 8 biocomparators with increasing peptide densities (i.e., peptide:liposome reaction ratios spanning 0, 8.5 × 10^−3^, 8.5 × 10^−2^, 8.5 × 10^−1^, 8.5, 85, 170, 255, 340 μmol/g) and validated their ability to sense input bacterial concentrations across 8 log units (0–10^8^ CFU/ml) using a fluorescent reporter (Fig. S5). To convert the release of signal proteases to a drug output, we designed protease-activatable prodrugs comprising cationic (polyarginine) anti-microbial peptides (AMP) (Fig. 3c and Table S2) in charge complexation with anionic peptide locks (polyglutamic acid)^55^ to block the activity of AMP. These drug-lock peptides were linked in tandem by OR gate peptides p_0_ and p_1_ (RTKR and ENLYFQG respectively) to allow signal proteases that directly cleave p_0_ or p_1_ to digitally control the output drug dose (Fig. 2). We designed one-third and two-thirds of the total drug dose to be unlocked by cleavage of p_0_ and p_1_, respectively, such that binary values 00, 01, 10, and 11 corresponded to 0/3, 1/3, 2/3, and 3/3 of the total drug dose (Fig. S6).

To confirm the therapeutic efficacy of our prodrug design, treatment of bacteria with locked drug had no significant cytolytic activity compared to untreated controls, but by contrast, treatment with protease-cleaved drug-lock complexes resulted in a significant reduction in bacterial colonies (Fig. 3d). We observed similar levels of bacterial cytotoxicity when AMP was directly loaded into liposomes, showing that charge complexation was required to fully block AMP activity (Fig. S5b). In human RBCs mixed with *E. coli* at concentrations ranging from 10^0^ to 10^9^ CFU/mL, samples containing a single biocomparator (*b*_0_) lacked the ability to eliminate bacteria as anticipated (output = 00). By contrast, increasing the number of biocomparators in the samples (*b*_0_ – *b*_3_) allowed our program to autonomously increase the drug dose (output 01, 10, and 11) in response to higher bacterial loads to completely eliminate infection burdens across nine orders of magnitude up to 10^9^ CFU/mL without increasing hemolysis (Fig. 3e and Fig. S7). Our data showed that biocircuits can be constructed using protease activity as a digital signal to execute autonomous drug delivery programs under a broad range of conditions.

### Controlled partitioning of shared resources as analog operations

**Fig. 4:**
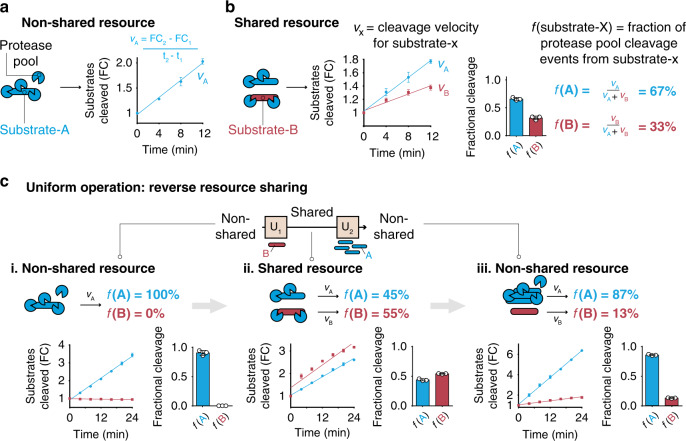
Controlling substrate concentration to tune resource sharing – the uniform operation. **a** One protease (blue pac man, Plasmin) cleaving one substrate (A, blue; 5FAM-KSVARTLLVK-(LysCPQ2)-C) represents a non-shared resource environment. The degree of substrate cleavage is quantified with cleavage velocity, ***v***_**A**_, (change in rate of substrate cleavage). The amount (i.e., moles) of cleaved substrate and is plotted as fold change (FC) from initial fluorescence at time = 0. **b** One protease (blue pac man) cleaving two substrates (substrate-A = blue, substrate-B = red = DABCYL-GPAALKAG-EDANS-R) represents a shared resource environment, as each substrate is competing for the same pool of proteases. The degree of resource sharing is quantified by the fractional cleavage score, ***f***(**X**), which estimates the fraction of cleavage events that resulted from substrate-X. This is calculated by normalizing the cleavage velocities for each substrate. c Implementation of the biological uniform operation on a two-state analog protease signal showing raw, kinetic fluorescence data over time (line graphs) used to calculate the fractional cleavage scores (bar graphs) A protease (Thrombin), first only exposed to the state-A substrate (5FAM-KTTGGRIYGG-(LysCPQ2)), is then exposed to the state-B substrate (i.e., U_1_, DABCYL-GPLGL-(DAP)-ARG-EDANS), resulting in equal fractions of the protease pool occupying either substrate state (~45 and 55% respectively). A second U-operation (U_2_), performed by adding state-A substrate in large molar excess, reversed the operation to restore the protease pool to its original state, which is that nearly the entire protease pool is cleaving substrate-A (~87%). For (**a**–**c**, data is presented as mean ± standard deviation (*n* = 3 biologically independent samples). Source data are provided as a Source data file.

We next sought to demonstrate that proteases in a shared resources environment can be used to build analog biocircuits. We first considered one pool of proteases (plasmin) cleaving one target peptide (substrate-A, blue) as representative of a non-shared resources environment (Fig. 4a). This substrate (5FAM-KSVARTLLVK-(LysCPQ2)-C) contained a fluorophore-quencher pair (5FAM and LysCPQ2, respectively) to allow quantification of the substrate cleavage velocity, v_A_, by monitoring increases in sample fluorescence over time. To construct a shared resource environment, we incubated plasmin with substrate-A and a second peptide, substrate-B (red), and observed that this caused total plasmin cleavage activity to be partitioned between substrate-A and substrate-B (Fig. 4b). To assess the degree of partitioning, we quantified the fractional cleavage score ***f*** (**substrate**−**X**), which reflects the average fraction of the protease pool cleavage events resulting from substrate-X. Fractional cleavage is analogous to fractional occupancy that is commonly used in models of receptor-ligand binding kinetics^56,57^. Here rather than measuring the fraction of receptors bound to a particular ligand, we measure the fraction of protease cleavage events bound to a particular substrate by quantifying the normalized cleavage velocities (Eq. 1).

1 $$f\left( {\mathrm{A}} \right) = \frac{{{\mathrm{products}}\,{\mathrm{formed}}\,{\mathrm{from}}\,{\mathrm{substrate}} - {\mathrm{A}}}}{{{\mathrm{total}}\,{\mathrm{products}}\,{\mathrm{formed}}}} = \frac{{v_A}}{{v_A + v_B}}$$

This fractional cleavage score quantifies the degree of resource sharing since when values of ***f***(**X**) approach 1, proteases are cleaving a single peptide (i.e., resource sharing is OFF). Conversely, when protease activity is uniformly partitioned between two substrates, the value of ***f***(**X**) approaches 0.5 (i.e., uniform resource sharing = ON).

To construct an operation capable of turning resource sharing ON and OFF, we developed the uniform (U) operation. The U operation is a two-step procedure (i.e., denoted by U_1_ and U_2_, respectively) that first takes as input a pool of proteases cleaving substrate-A in a non-shared resource setting (Fig. 4c i) and outputs a state where protease activity is partitioned uniformly between substrate-A and substrate-B (i.e., ***f***(**A**) = ***f***(**B**) = 50%). This was done experimentally by adding substrate-B such that the fractional cleavage scores were equivalent (Fig. 4c ii). The second half of this operation (U_2_) functions to reverse, or turn resource sharing OFF. This was done by adding a large molar excess of substrate-A such that the fractional cleavage score for substrate-A would approach 100% (***f***(**A**) = 87%, *n* = 3) (Fig. 4c iii). These results demonstrated that by controlling the available concentration of two substrates, resource sharing can be turned ON and OFF in a pool of proteases.

**Fig. 5:**
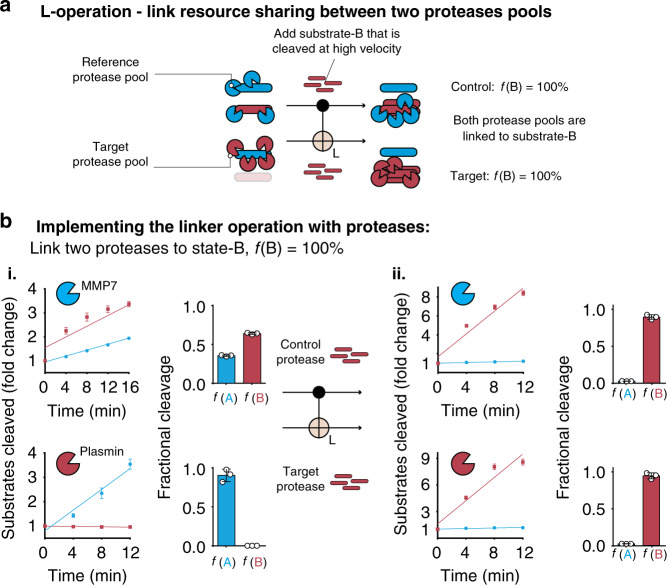
Controlling substrate concentration to match two protease pools to the same state of shared resources—the linker operation. **a** Schematic depicting the biological implementation of the L-operation. The state-B substrate (red) is added to two different protease signals (blue = control, red = target) at such a concentration that the output state-B substrate fractional cleavage (i.e., ***f***(**B**)) approaches 100%. **b** Experimental example of the L-operation. The output states of control protease pool (MMP7, top row) and target protease pool (plasmin, bottom row) are matched by addition of the state-B substrate (DABCYL-GPAALKAG-EDANS-R) such that the output protease populations have a matched fractional cleavage close to 100% (97% and 98%). The amount (i.e., moles) of cleaved substrate and is plotted as fold change (FC) from initial fluorescence at time = 0. Data is presented as mean ± standard deviation (*n* = 3 biologically independent samples). Source data are provided as a Source data file.

Next, we sought to build an operation with a 2-input/2-output format. Operations with multiple inputs and outputs are important because they enable the comparison or integration of different signals, such as a Half Adder circuit or 2-bit Synchronous counter. For our system, we designed an operation called the Linker operation that functions by matching the shared resources state for two input protease signals, named the reference and target signal, to a non-shared state. Here if the reference protease is in a state of shared resources, the Linker operation then matches (i.e., links) the fractional cleavage scores of the reference and target proteases to ***f***(**B**) = 100% (Fig. 5a). To demonstrate the L-operation experimentally, we started with a reference protease pool (i.e., MMP7) initially in a state of shared resources (i.e., ***f***(**A**) = ***f***(**B**) = 50%) and a target protease pool (i.e., plasmin) in a state of non-shared resources (i.e., ***f***(**A**) = 100%). Then, we applied the L-operation to match the output states of the reference protease pool (i.e., MMP7) and the target protease pool (i.e., plasmin) by adding the state-B substrate (DABCYL-GPAALKAG-EDANS-R). We observed that the output protease pools were both matched to a state of non-shared resources with fractional cleavage scores approaching 100% for substrate-B (i.e., reference ***f***(**B**) = 97%, target ***f***(**B**) = 98%) (Fig. 5b). These results demonstrated that by controlling the concentration of the state-B substrate, the degree of resource sharing can be matched between protease pools.

### Solving a mathematical problem with proteases and shared resources

**Fig. 6:**
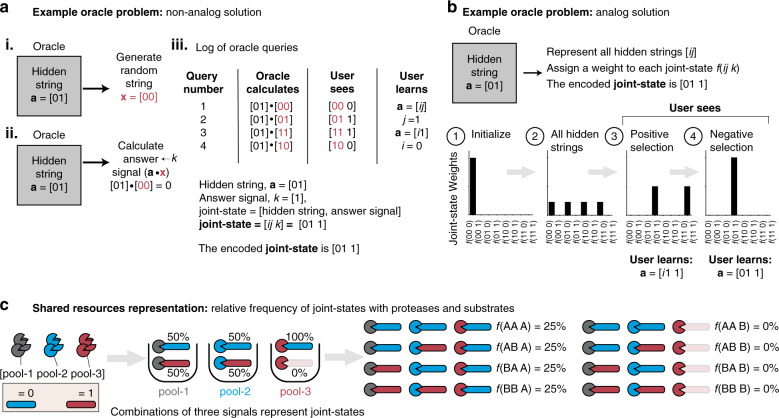
Proteases in shared resource environments represent joint-states in the oracle problem. **a** Schematic of non-analog solution to oracle problem. The oracle problem is a general inference problem where an oracle (i.e., gray box) is hiding a string of digits (i.e., hidden string **a** = [***ij***]). The goal is to infer the value of the hidden string. To accomplish this, the user can make oracle queries, which cause the oracle to (1) generate a random string (red text, *x*) and (2) take the dot product (i.e., scalar product of two vectors) of the hidden and random strings to produce the answer signal, *k*. **b** Schematic showing conceptually how to solve the oracle with proteases. To set up the problem, (1) the user first initializes all variables to 0 (i.e., [***ij k***] = [00 0], ***f*** = (000) = 100%). (2) Then, the user represents all hidden strings simultaneously (i.e., ***f*** = (000) = ***f*** = (010) = ***f*** = (100) = ***f*** = (110) = 25%). (3) The user applies analog operations to learn which digit(s) in the hidden string is/are matched to the answer signal (i.e., positive selection). (4) The user validates that the remaining digit(s) in the hidden string is/are not matched to the answer signal (i.e., negative selection). **c** Schematic demonstrating how the weights for all eight joint-states are calculated using the fractional cleavage scores of individual protease pools. Here, uniform resource sharing is ON (i.e., cleaving substrates A and B equally, or ***f***(**A**) = ***f***(**B**) = 50%) for the two hidden string proteases (gray and blue proteases) and resource sharing is OFF (i.e., ***f***(**A**) = 100%) and ***f***(**B**) = 0%)) for the answer protease (red protease). The weights for all eight possible joint-states (i.e., ***f***(**AAA**), ***f***(**AAB**), …, ***f***(**BBB**)) are calculated by multiplying the fractional cleavage scores for each of the three individual protease pools (e.g., ***f***(**AAA**) = 25%), and these weights will collectively add up to 100%.

We designed the Uniform and Linker operations based on previously described analog operations that could be used to solve one type of oracle problem, the LPN^58^. Here an oracle contains a hidden string, **a**, of digits (Fig. 6a represents the case where the hidden string **a** = [01]) that is unknown to the user. To infer the value of the hidden string, the user can make "oracle queries" that cause the oracle to generate random strings, **x**, and return the dot product (i.e., the scalar product of two vectors) between the hidden string and the random string (i.e., **a**·**x**) as an answer signal, ***k***. Conventionally, the possible hidden strings (i.e., [00], [01], [10], [11]) and answer signals (i.e., [0] or [1]) are concatenated to form a 3-valued joint-state (i.e., [hidden string, answer signal] = [***ij k***]) that has eight possible permutations (i.e., [00 0], [00 1], …, [11 1]). By querying the oracle and eliminating hidden strings that could not have produced the observed answer signal, the user can infer the value of the hidden string. For example, if on query number-1 the oracle generates the random string x = [00], then the user will see the answer signal = 0 (i.e., **a**·**x** = [01]·[00] = 0), which does not provide any information about the hidden string *a*, as it can take on any value. However, if on query number-2 the oracle generates the random string x = [01], then the user will observe that the answer signal = 1 (i.e., **a**·**x** = [01]·[01] = 1) and can determine that 1 is the second-digit (i.e., ***j*** = 1) (Fig. 6a). By repeating oracle queries, the user will be able to eventually infer the value of the hidden string. However, each query yields at most one additional piece of information.

By contrast, rather than querying the oracle in a stepwise manner, analog circuits are designed to test multiple possible joint-states simultaneously by assigning each a relative weight (e.g., ***f***(000), ***f***(001), …, ***f***(111)). This approach allows the user to learn one or more piece of information after each operation. For example, a non-biological algorithm of the oracle problem^58^ starts by initializing all values in the joint-state to 0 (i.e., ***f***(***ijk***) = ***f***(000) = 100%), and then simultaneously represents all possible hidden strings (i.e., ***f***(000) = ***f***(010) = ***f***(100) = ***f***(110) = 25%) (Fig. 6b; steps 1 and 2). Then, to solve the oracle, the user applies an analog operation to learn which digits in the hidden string are matched with the answer signal (i.e., positive selection) and then validates that the remaining digits in the hidden string are not matched with the answer signal (i.e., negative selection) (Fig. 6b; steps 3 and 4, respectively). For example, in the case where the hidden string is [01], the correct joint-state is [01 1]. Therefore, after positive selection, the remaining joint-states [01 1] and [11 1] are equally weighted (i.e., ***f***(011) = ***f***(111) = 50%). After negative selection, the user is able to determine that the correct joint-state with the highest weight is [01 1] (i.e., ***f***(011) = 100%).

Based on this previously described analog algorithm, we next sought to show that proteases can be used to implement and solve the oracle problem. When a protease is in a shared resource environment with two substrates (i.e., blue and red; Fig. 6c), its fractional cleavage scores ***f***(**A**)and ***f***(**B**) take on a value between 0% and 100%, which is analogous to relative weights. Therefore, if two proteases are designated to represent possible hidden string values (gray and blue) and one protease (red) to represent the answer signal, this system of three proteases can then represent all eight possible joint-states simultaneously. For example, in the setting where uniform resource sharing is ON for the two hidden string proteases (i.e., gray and blue protease each cleaving substrates A and B equally, or ***f***(**A**) = ***f***(**B**) = 50%) and resource sharing for the answer protease is OFF (i.e., red protease; ***f***(**A**) = 100% and ***f***(**B**) = 0%), then the weights for all eight possible joint-states (i.e., ***f***(**AAA**), ***f***(**AAB**), …, ***f***(**BBB**)) can be individually calculated by multiplying the fractional cleavage scores of the three proteases (e.g.,., ***f***(**AAA**) = 25%) (Fig. 6c). These analog weights for the joint-states would always total to 100%, and its values would depend on the extent of resource sharing for each of the three proteases.

**Fig. 7:**
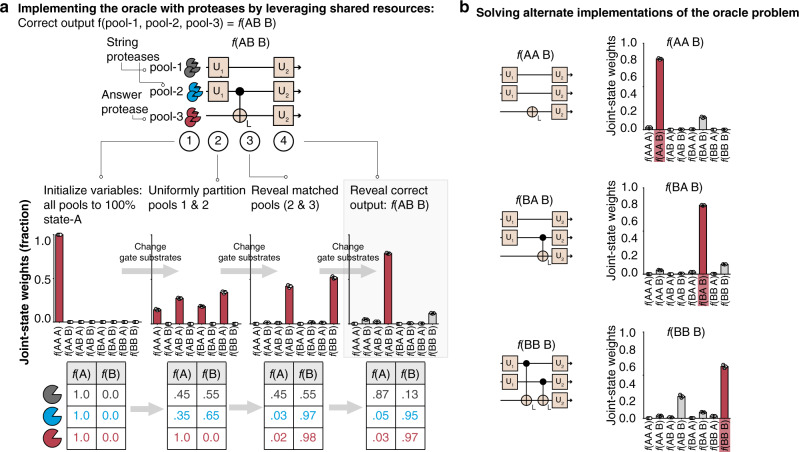
Using shared resources to represent a mathematical solution to the oracle problem. **a** (Top) Schematic representing the order in which the U_1_, U_2_, and L gates are applied to each of the three protease pools (pool-1 Gray = Thrombin, pool-2 Blue = MMP7 and pool-3 Red = Plasmin). Each line is specific to one protease, and the arrow indicates the order in which each gate was applied. (Bottom) Tables show the experimental data of the fractional cleavage values (i.e., ***f***(**A**) and ***f***(**B**)) for each pool of proteases (gray, blue, red). Bar graphs represent the relative weights of each joint-state after each round of substrate operations (steps 1–4), which were calculated using the fractional cleavage data in the corresponding table below. **b** Experimental data (bar graphs) showing the remaining three implementations of the oracle problem (i.e., ***f***(**AAB**), ***f***(**BAB**), and ***f***(**BBB**)). Schematics to the left of the bar graphs are the schematics representing the order in which the U_1_, U_2_, and L gates were applied for each problem. Bar graphs represent the relative weight for each of the eight possible output joint-states. Red bar indicates the correct joint-state with the highest weight. For **a**, **b**, data is presented as mean ± standard deviation (*n* = 3 biologically independent samples). Source data are provided as a Source data file.

Based on the non-biological implementation of the oracle problem^58^, we designed analog biocircuits using a combination of Uniform (i.e., U_1_ and U_2_) and Linker operations to solve the oracle for the particular joint-state ***f***(**AB B**) (Fig. 7a). All proteases were initialized to cleaving substrate-A (i.e., joint-state ***f***(**AAA**) = 100%)). Then to represent all hidden strings simultaneously (i.e., answer signal, ***k*** = **A**; ***f***(**AAA**) = ***f***(**ABA**) = ***f***(**BAA**) = ***f***(**BBA**) = 25%)), we applied U_1_ Uniform operations to protease pools 1 and 2 to create a state of shared resources within each pool (Fig. 7a; steps 1 and 2). Then, we applied L-operation(s) to reveal which protease pools in the hidden string were matched with the answer signal (i.e., positive selection) by outputting matched proteases to the same state of shared resources (i.e., pool-2 ***f***(**B**) = 100%, pool-3 ***f***(**B**) = 100%) (Fig. 7a; step 3). Lastly, we validated that the remaining protease pool (i.e., pool-1) was not matched with the answer signal (i.e., negative selection) by applying three U_2_ Uniform operations, which reversed pool-1 to a state of non-shared resources cleaving substrate-A (i.e., pool-1 ***f***(**A**) = 100%) (Fig. 7a; step 4). Experimentally, we found that this protease-based, shared resources solution yielded the correct joint-state with the highest weight (***f***(**ABB**) = 80%, *n* = 3) when compared to the seven other possible joint-states (each other answer <12%, *n* = 3) (Fig. 7a). To further support these results, we solved the remaining three implementations of the oracle problem (proteases and substrates in Table 1), which were ***f***(**AAB**), ***f***(**BAB**), and ***f***(**BBB**). In each of these three configurations, the protease-based solution outputted the correct joint-state with the highest weight (Fig. 7b).

**Table 1:** Legend to identify the proteases and substrates used in all four oracle problems.

Oracle	Pool	Protease	Substrate-A	Substrate-B
f(AA B)	Pool-1	Thrombin	**5FAM**-KTTGGRIYGG-(**LysCPQ2**)-C	**DABCYL**-GPLGL-(**DAP**)-ARG-**EDANS**-R
f(AA B)	Pool-2	C1s	**5FAM**- KYLGRSYKV -(**LysCPQ2**)-C	**DABCYL**-GPAALKAG-**EDANS**-R
f(AA B)	Pool-3	Plasmin	**5FAM**-KSVARTLLVK-(**LysCPQ2**)-C	**DABCYL**-GPAALKAG-**EDANS**-R
f(AB B)	Pool-1	Thrombin	**5FAM**-KTTGGRIYGG-(**LysCPQ2**)-C	**DABCYL**-GPLGL-(**DAP**)-ARG-**EDANS**-R
f(AB B)	Pool-2	MMP7	**5FAM**-KYLGRSYKV-(**LysCPQ2**)-C	**DABCYL**-GPAALKAG-**EDANS**-R
f(AB B)	Pool-3	Plasmin	**5FAM**-KSVARTLLVK-(**LysCPQ2**)-C	**DABCYL**-GPAALKAG-**EDANS**-R
f(BA B)	Pool-1	MMP7	**5FAM**-KYLGRSYKV-(**LysCPQ2**)-C	**DABCYL**-GPAALKAG-**EDANS**-R
f(BA B)	Pool-2	Thrombin	**5FAM**-KTTGGRIYGG-(**LysCPQ2**)-C	**DABCYL**-GPLGL-(**DAP**)-ARG-**EDANS**-R
f(BA B)	Pool-3	Plasmin	**5FAM**-KSVARTLLVK-(**LysCPQ2**)-C	**DABCYL**-GPAALKAG-**EDANS**-R
f(BB B)	Pool-1	MMP7	**5FAM**-KYLGRSYKV-(**LysCPQ2**)-C	**DABCYL**-GPAALKAG-**EDANS**-R
f(BB B)	Pool-2	Cathepsin G	**5FAM**-KSVARTLLVK-(**LysCPQ2**)-C	**DABCYL**-GPAALKAG-**EDANS**-R
f(BB B)	Pool-3	Plasmin	**5FAM**-KSVARTLLVK-(**LysCPQ2**)-C	**DABCYL**-GPAALKAG-**EDANS**-R

Non-bold, capital letters represent single-letter amino acid codes. Bold letters represent other functional groups: **DABCYL** = 4-(dimethylaminoazo)benzene-4-carboxylic acid; EDANS = (5-((2-Aminoethyl)amino)naphthalene-1-sulfonic acid); **LysCPQ2** = lysine-conjugated quencher (**CPC** Scientific); **DAP** = 2-3, diaminopropionic acid.

## Discussion

By interpreting protease activity as carrying digital or analog information, we demonstrated the use of proteases in molecular biocircuits. We used two-valued protease operations carried out by Boolean logic gates to construct an ADC as an autonomous drug delivery biocircuit to clear blood of bacteria across nine orders magnitude in concentration. To construct our biological ADC, we designed biocomparators using peptide-caged liposomes because these materials are well-tolerated and biologically compatible^59,60^. Our molecular approach is distinct from existing synthetic protein^36^ and genetic circuit^48,61^ methods that require protein or organismal engineering to control signaling, including the non-trivial OFF state for proteases which has required insertion strategies^20–22^ artificial autoinhibitors^36^, or dimerizing leucine zippers^48^ to control. Parallel to our use of liposome-based particles to control protease activity, liposomes have also been used in past studies such as synthetic minimal cells to control the expression of genetic circuits by liposome fusion^49,62^. Our approach may be amenable to integration with these genetic approaches, if for example, these circuits were redesigned to input or output (i.e., express) proteases.

In addition to manipulating exclusively two-valued protease operations, we demonstrated the use of proteases in a shared resources environment to represent analog operations. Analog biocircuits^24,63^ have been demonstrated in systems including gene circuits, DNA strand displacement cascades, and restriction enzymes^27–29,45^. Many of these circuits^23,63–65^ primarily process continuous signals via summing, thresholding, and binning using comparators^28^ or linear threshold circuits^29^ (i.e., multi-input comparator). A few studies have used analog biocircuits to represent and operate on multiple states simultaneously by assigning a weight to each state (i.e., fuzzy logic). As an example, a pool of restriction enzymes, *FokI*, was partitioned between different DNA substrates to represent two output states with a weight (i.e., 0 → 100%) calculated by the relative abundance of each DNA product (i.e., 16 versus 17 nt-long product bands)^45^. By comparison, we used promiscuous proteases to create a shared resources environment where one protease is partitioned between cleaving two peptide substrates. In the context of the oracle problem, we used three pools of proteases in a state of shared resources to represent eight output states simultaneously. The strategy of representing multiple states simultaneously enables problems that typically require an iterative approach (i.e., each possible answer is tested sequentially) to be solved more efficiently, which has been demonstrated using strand displacement cascades to solve combinatorial math problems^15^. Looking forward, this strategy may have advantages for implementing biological problems that require a type of iterative approach, such as the decryption of gene circuits^66^.

## Methods

### Protease cleavage assays

All protease cleavage assays were performed with a BioTek Cytation 5 Imaging Plate Reader, taking fluorescent measurements at 485/528 nm and 540/575 nm (excitation/emission) for read-outs measuring peptide substrates terminated with FITC (Fluorescein isothiocyanate) and 5-TAMRA (5-Carboxytetramethylrhodamine), respectively. Kinetic measurements were taken every minute over the course of 60–120 min at 37 C. West Nile Virus NS3 protease (WNVp) and Tobacco Etch Virus protease (TEVp), along with their substrates, inhibitors and buffers were obtained from Anaspec, Inc. (Fremont, CA). Phospholipase C (PLC), Phosphatidylinositol-Specific (from Bacillus cereus) was purchased from Thermo Fisher Scientific (Waltham, MA). Activity RFU measurements were normalized to time 0 measurement, and as such later time points (after time-0) represent fold change in signal. Granzyme B (GzmB) was purchased from PeproTech, Inc. (Rocky Hill, NJ). Thrombin and Factor XIa were purchased from Haematologic Technologies (Essex, VT). Outer Membrane Protease T (OmpT, Protease 7) was purchased from Lifespan Biosciences (Seattle, WA). C1r was purchased from Millipore Sigma (Burlington, MA). GzmB, Thrombin, Factor XIa, and C1r fluorescent peptide substrates were custom ordered from CPC Scientific (Sunnyvale, CA). OmpT fluorescent peptide substrate was custom ordered from Genscript (Piscataway, NJ). See Tables S1 and S2 for more information regarding proteases, substrates, peptides, and inhibitors.

In all, 10 μL of liposomes (34 mM lipids) loaded with TEVp (1 μg protease/17 mmol liposome) were coincubated with 50 μL TEVp substrate in provided activity buffer (pH 7.5). In all, 2 μL of PLC (100 U/mL) was added to the experimental group, and 2 μL of assay buffer was added to the control group (Fig. 2b).

In all, 10 μL of liposomes (34 mM lipids) loaded with TEVp (1 μg protease/17 mmol liposome), embedded with 10 mol% CPAA and crosslinked at 0.1% efficiency with GzmB substrate were coincubated with 50 μL TEVp substrate in provided activity buffer (pH 7.5). In all, 2 μL PLC (100 U/mL) was added to both the control and experimental group. In all, 2 μL GzmB (0.1 μg/uL) was added only to experimental group (Fig. 2c).

All amounts of protease, substrate, and inhibitor for WNVp and TEVp were added according to instructions from Anaspec WNVp and TEVp activity kit. All conditions incubated with WNVp inhibitor include protease of interest incubated with its primary substrate. GzmB was added at a working concentration of (0.01 mg/mL) to 2 μM of its peptide substrate (Fig. 2d).

All four biocomparator levels (*b*_0_ – *b*_3_, 50 mM lipids each) were added together (10 μL each), and co-incubated with 13 μL of GzmB solution (concentration varies depending on condition), 2 μL of PLC (100 U/mL), 0.5 μL of WNVp substrate (after diluted 100x according to manufacturer’s instructions), 0.5 μL of TEVp substrate (after diluted 100x according to manufacturer’s instructions), and 4 μL of assay buffer. Biocomparator levels 0–3 are referenced by peptide cage crosslinking efficiencies of 0%, 0.01%, 1%, and 100%, respectively. Plotted values are taken at minute 30 and normalized to starting values (time 0, or equivalently, the no protease control). Unpaired, one-way *t*-tests (*n* = 4) were performed between the condition with GzmB and the negative control (no GzmB) for each respective output (i.e., p_0_ or p_1_) (Fig. 2e).

For recombinant OmpT condition, 2 μL of OmpT (0.5 mg/mL) was added to 18 μL of 2 μM OmpT substrate. For *E. coli* condition, 2 μL of *E. coli* (10^9^ CFU/mL) was added to 18 μL of 2 μM OmpT substrate. In all, 2 μL of DI H_2_O was added to the negative control, along with 18 μL of OmpT substrate (Fig. 3b).

For Fig. 4a, the protease Plasmin (250 nM) was incubated with substrate-A (5FAM-KSVARTLLVK-(LysCPQ2)-C; concentration = 0.24 μM) in PBS. For Fig. 4 b, the protease Plasmin (250 nM) was incubated with substrate-A (5FAM-KSVARTLLVK-(LysCPQ2)-C; concentration = 0.24 μM) as well as substrate-B (DABCYL-GPAALKAG-EDANS-R; concentration = 0.2 μM) simultaneously in PBS. Experiments were read on Plate Reader at 37 C with plate sealer and plastic plate cover (Fig. 4a, b).

For Fig. 4c panel i, the protease Thrombin (250 nM) was incubated with substrate-A (5FAM-KTTGGRIYGG-(LysCPQ2)-C; concentration = 0.64 μM) in PBS. For panel ii, the protease Thrombin (250 nM) was incubated with substrate-A (5FAM-KTTGGRIYGG-(LysCPQ2)-C; concentration = 0.64 μM) as well as substrate-B (DABCYL-GPLGL-(DAP)-ARG-EDANS-R; concentration = 6.75 μM) simultaneously in PBS. For panel iii, the protease Thrombin (250 nM) was incubated with substrate-A (5FAM-KTTGGRIYGG-(LysCPQ2)-C; concentration = 1.28 μM) as well as substrate-B (DABCYL-GPLGL-(DAP)-ARG-EDANS-R; concentration = 6.75 μM) simultaneously in PBS. Experiments were read on Plate Reader at 37 C with plate sealer and plastic plate cover (Fig. 4c).

For Fig. 5b panel i, the protease MMP7 (250 nM) was incubated with substrate-A (5FAM-KYLGRSYKV-(LysCPQ2)-C; concentration = 2.53 μM) as well as substrate-B (DABCYL-GPAALKAG-EDANS-R; concentration = 29.90 μM) simultaneously in PBS. The protease Plasmin (250 nM) was incubated with substrate-A (5FAM-KSVARTLLVK-(LysCPQ2)-C; concentration = 0.24 μM) in PBS. For panel II, the protease MMP7 (250 nM) was incubated with substrate-A (5FAM-KYLGRSYKV-(LysCPQ2)-C; concentration = 2.53 μM) as well as substrate-B (DABCYL-GPAALKAG-EDANS-R; concentration = 149.50 μM) simultaneously in PBS. The protease Plasmin (250 nM) was incubated with substrate-A (5FAM-KSVARTLLVK-(LysCPQ2)-C; concentration = 0.24 μM) as well as substrate-B (DABCYL-GPAALKAG-EDANS-R; concentration = 74.75 μM) simultaneously in PBS (Fig. 5b).

For Fig. 7a, Oracle ***f***(**AB B**), graph (1), the protease Thrombin (250 nM) was incubated with substrate-A (5FAM-KTTGGRIYGG-(LysCPQ2)-C; concentration = 0.64 μM) in PBS. The protease MMP7 (250 nM) was incubated with substrate-A (5FAM-KYLGRSYKV-(LysCPQ2)-C; concentration = 2.53 μM) in PBS. The protease Plasmin (250 nM) was incubated with substrate-A (5FAM-KSVARTLLVK-(LysCPQ2)-C; concentration = 0.24 μM) in PBSg (Fig. 7a, b).

For Fig. 7a, Oracle *f*(AB B), graph (2), the protease Thrombin (250 nM) was incubated with substrate-A (5FAM-KTTGGRIYGG-(LysCPQ2)-C; concentration = 0.64 μM) as well as substrate-B (DABCYL-GPLGL-(DAP)-ARG-EDANS-R; concentration = 6.75 μM) simultaneously in PBS. The protease MMP7 (250 nM) was incubated with substrate-A (5FAM-KYLGRSYKV-(LysCPQ2)-C; concentration = 2.53 μM) as well as substrate-B (DABCYL-GPAALKAG-EDANS-R; concentration = 29.90 μM) simultaneously in PBS. The protease Plasmin (250 nM) was incubated with substrate-A (5FAM-KSVARTLLVK-(LysCPQ2)-C; concentration = 0.24 μM) in PBS.

For Fig. 7a, Oracle *f*(AB B), graph (3), the protease Thrombin (250 nM) was incubated with substrate-A (5FAM-KTTGGRIYGG-(LysCPQ2)-C; concentration = 0.64 μM) as well as substrate-B (DABCYL-GPLGL-(DAP)-ARG-EDANS-R; concentration = 6.75 μM), plus an additional substrate-B for the Linker operation (DABCYL-GPAALKAG-EDANS-R; concentration = 29.90 μM), simultaneously in PBS. The protease MMP7 (250 nM) was incubated with substrate-A (5FAM-KYLGRSYKV-(LysCPQ2)-C; concentration = 2.53 μM) as well as substrate-B (DABCYL-GPAALKAG-EDANS-R; concentration = 29.90 μM) plus an additional substrate-B for the Linker operation (DABCYL-GPAALKAG-EDANS-R; concentration = 119.60 μM), simultaneously in PBS. The protease Plasmin (250 nM) was incubated with substrate-A (5FAM-KSVARTLLVK-(LysCPQ2)-C; concentration = 0.24 μM) plus an additional substrate-B for the Linker operation (DABCYL-GPAALKAG-EDANS-R; concentration = 74.75 μM), in PBS.

For Fig. 7a, Oracle *f*(AB B), graph (4), the protease Thrombin (250 nM) was incubated with substrate-A (5FAM-KTTGGRIYGG-(LysCPQ2)-C; concentration = 1.28 μM) as well as substrate-B (DABCYL-GPLGL-(DAP)-ARG-EDANS-R; concentration = 6.75 μM), plus an additional substrate-B for the Linker operation (DABCYL-GPAALKAG-EDANS-R; concentration = 29.90 μM), simultaneously in PBS. The protease MMP7 (250 nM) was incubated with substrate-A (5FAM-KYLGRSYKV-(LysCPQ2)-C; concentration = 5.07 μM) as well as substrate-B (DABCYL-GPAALKAG-EDANS-R; concentration = 29.90 μM) plus an additional substrate-B for the Linker operation (DABCYL-GPAALKAG-EDANS-R; concentration = 119.60 μM), simultaneously in PBS. The protease Plasmin (250 nM) was incubated with substrate-A (5FAM-KSVARTLLVK-(LysCPQ2)-C; concentration = 0.48 μM) plus an additional substrate-B for the Linker operation (DABCYL-GPAALKAG-EDANS-R; concentration = 74.75 μM), in PBS.

For Fig. 7b, Oracle *f*(AA B) (i.e., graph 4), the protease Thrombin (250 nM) was incubated with substrate-A (5FAM-KTTGGRIYGG-(LysCPQ2)-C; concentration = 1.28 μM) as well as substrate-B (DABCYL-GPLGL-(DAP)-ARG-EDANS-R; concentration = 6.75 μM), plus an additional substrate-B for the Linker operation (DABCYL-GPAALKAG-EDANS-R; concentration = 29.90 μM), simultaneously in PBS. The protease Complement protease C1s (250 nM) was incubated with substrate-A (5FAM- KYLGRSYKV -(LysCPQ2)-C; concentration = 0.24 μM) as well as substrate-B (DABCYL-GPAALKAG-EDANS-R; concentration = 29.90 μM) plus an additional substrate-B for the Linker operation (DABCYL-GPAALKAG-EDANS-R; concentration = 29.90 μM), simultaneously in PBS. The protease Plasmin (250 nM) was incubated with substrate-A (5FAM-KSVARTLLVK-(LysCPQ2)-C; concentration = 0.48 μM) plus an additional substrate-B for the Linker operation (DABCYL-GPAALKAG-EDANS-R; concentration = 74.75 μM), in PBS.

For Fig. 7b, Oracle ***f***(**BA B**) (i.e., graph 4), the protease MMP7 (250 nM) was incubated with substrate-A (5FAM-KYLGRSYKV-(LysCPQ2)-C; concentration = 5.07 μM) as well as substrate-B (DABCYL-GPAALKAG-EDANS-R; concentration = 29.90 μM) plus an additional substrate-B for the Linker operation (DABCYL-GPAALKAG-EDANS-R; concentration = 119.60 μM), simultaneously in PBS. The protease Thrombin (250 nM) was incubated with substrate-A (5FAM-KTTGGRIYGG-(LysCPQ2)-C; concentration = 1.28 μM) as well as substrate-B (DABCYL-GPLGL-(DAP)-ARG-EDANS-R; concentration = 6.75 μM), plus an additional substrate-B for the Linker operation (DABCYL-GPAALKAG-EDANS-R; concentration = 29.90 μM), simultaneously in PBS. The protease Plasmin (250 nM) was incubated with substrate-A (5FAM-KSVARTLLVK-(LysCPQ2)-C; concentration = 0.48 μM) plus an additional substrate-B for the Linker operation (DABCYL-GPAALKAG-EDANS-R; concentration = 74.75 μM), in PBS.

For Fig. 7b, Oracle ***f***(**BB B**) (i.e., graph 4), the protease MMP7 (250 nM) was incubated with substrate-A (5FAM-KYLGRSYKV-(LysCPQ2)-C; concentration = 5.07 μM) as well as substrate-B (DABCYL-GPAALKAG-EDANS-R; concentration = 29.90 μM) plus an additional substrate-B for the Linker operation (DABCYL-GPAALKAG-EDANS-R; concentration = 119.60 μM), simultaneously in PBS. The protease Cathepsin G (250 nM) was incubated with substrate-A (5FAM-KSVARTLLVK-(LysCPQ2)-C; concentration = 0.05 μM) as well as substrate-B (DABCYL-GPAALKAG-EDANS-R; concentration = 29.90 μM), plus an additional substrate-B for the Linker operation (DABCYL-GPAALKAG-EDANS-R; concentration = 29.90 μM), simultaneously in PBS. The protease Plasmin (250 nM) was incubated with substrate-A (5FAM-KSVARTLLVK-(LysCPQ2)-C; concentration = 0.48 μM) plus an additional substrate-B for the Linker operation (DABCYL-GPAALKAG-EDANS-R; concentration = 74.75 μM), in PBS.

### Liposome synthesis and characterization

Liposome synthesis kit, PIPES buffer, EDC*MeI, and spin filters (100 kDa m.w.c.o. filters) were purchased from Millipore Sigma (Burlington, MA). Cholesterol-anchored Polyacrylic Acid (4400 g/mol, 30–40 COOH groups/molecule, structure in Fig. S2a) was custom ordered from Nanocs (Boston, MA). Float-a-lyzer dialysis tubes (100 kDa m.w.c.o., 1 mL) were purchased from Spectrum Labs (Rancho Dominguez, CA). Synthesis protocol is adapted from the methods used by Basel et. al. Liposomes were loaded with respective protease inhibitor cocktail amounts, and concentration was estimated via absorbance. Standard curve for estimating concentration of liposomes was used by correlating absorbance of liposome solution at 230 nm with known standard concentrations. CPAA was vortexed in warm water (<10 mg/mL) and volume was added such that there was 10 mol% CPAA relative to the molarity of lipids in the liposome solution. Solution was incubated for 1 h at room temperature, or overnight at 4 C. Excess polymer and materials were removed via centrifugation (spin filters, 3–5 times at 4700 × *g* for 10 min) or float-a-lyzer membranes (4 °C in spinning water overnight). EDC*MeI was dissolved into 10 mM PIPES buffer and volume was added such that EDC*MeI:CPAA ratio was 4:1. Solution was incubated for 20 min at room temperature. Excess EDC was filtered out via centrifugation or dialysis tubes. Peptide crosslinker was added at desired molar ratio and incubated for 1 hour at room temperature or overnight at 4 C. Excess peptide was filtered via centrifugation or dialysis tubes. Change in liposome hydrodynamic diameter was measured via DLS on a Zetasizer Nano ZS, Malvern Panalytical (Netherlands). Volumes loaded into biocomparators include concentrations of proteases and inhibitors as follows: *b*_0_ = empty; *b*_1_ = 20 μL WNVp (0.1 mg/mL) + 80 μL DI H_2_O; *b*_2_ = 50 μL WNV inhibitor (1 μM) + 50 μL TEVp (0.04 mg/mL); *b*_3_ = 50 μL WNVp (0.1 mg/mL) + 50 μL TEVp (0.08 mg/mL).

### Bacterial cytotoxicity and human red blood cell hemolysis assays

Bacterial culture and cytotoxicity measurement. DH5α *Escherichia coli* were a gift from Todd Sulchek’s BioMEMS lab at Georgia Tech. *E. coli* were cultured in LB broth (Lennox) at 37 °C and plated on LB agar (Lennox) plates. LB broth was purchased from Millipore Sigma (Burlington, MA) and LB agar was purchased from Invitrogen (Carlsbad, CA). AMP and locked AMP were custom ordered from Genscript (Piscataway, NJ). See Table S1 for more information. Bacteria were grown to a concentration of 10^9^ CFU/mL before being used for experiments. Concentration was estimated by measuring the OD_600_ of the bacterial suspension, where an OD_600_ of 1.000 corresponds to a concentration of 8 × 10^8^ CFU/mL. Bacterial cell viability was measured by making eight 10-fold serial dilutions, and plating three 10 μL spots on an LB agar plate. Plates were incubated overnight at 37 °C, and CFUs were counted. Untreated bacteria CFU counts served as control for 0% cytotoxicity, and bacteria + IPA (or 0 countable CFUs) served as control for 100% cytotoxicity.

RBC collection and hemolysis measurement. Healthy blood donors had abstained from aspirin in the last two weeks, and written informed consent was obtained according to GT IRB H15258. Blood was drawn by median cubital venipuncture into sodium citrate (3.2%). The sample was subsequently centrifuged at 150 × *g* for 15 min, and the resulting platelet rich plasma was discarded. Red blood cells were then washed three times with phosphate buffered saline (PBS). For each wash, 12 mL of PBS were added, the sample was centrifuged at 200 × *g* for 10 min, and the supernatant was discarded. Hemolysis was estimated by spinning down experimental RBC samples and measuring the absorbance of the supernatant at 450 nm. Absorbance values corresponding to 100% hemolysis came from incubating RBCs with 0.1% Tween-20. Absorbances corresponding to 0% hemolysis came from untreated RBCs.

For bacterial cytotoxicity measurements, 25 μL of antimicrobial peptide (AMP) was added, pertaining to 7 concentrations ranging between 7.6 nM and 7.6 mM. In all, 20 μL of bacteria (10^7^ CFU/mL) were added, and the sample was filled to 200 μL with LB broth in PCR tubes. Sample tubes were taped on a plate shaker (250 RPM) incubating at 37 C for 8 h. For RBC hemolysis measurements, the same assay was performed, but used 20 μL of donor RBCs instead of bacteria solution (Fig. 3c).

For bacteria only condition, 5 μL of bacteria (10^9^ CFU/mL) were added to 95 μL LB broth. For bacteria + AMP p_1_, 58 μL of AMP p_1_ (1.7 mM) were added to 5 μL of bacteria (10^9^ CFU/mL), with the solution being filled to 100 μL with LB broth. For bacteria + protease + locked AMP p_1_, 20 μL of TEVp (4 μg/mL) and 58 μL of AMP p_1_ (1.7 mM) were added to 5 μL of bacteria (10^9^ CFU/mL), with the solution being filled to 100 μL with LB broth. Samples in PCR tubes were taped to a plate shaker (250 RPM) incubating at 37 C for 1 h. Serial dilutions and plating were then performed to measure viable bacteria concentrations (Fig. 3d).

Each condition includes 20 μL of the bioprogram (2 μL of PLC, 6 μL D_1_, 6 μL D_2_, 6 μL D_3_), 20 μL of bacteria, 10 μL of RBCs, 24 μL of locked peptide drug (9 μL of 1.7 mM AMP p_1_ and 15 μL of 0.53 mM AMP p_0_), and 126 μL PBS. The concentration of bacteria, and the presence of each biocomparator, depends on the experimental condition (Fig. 3e). Samples in PCR tubes were taped to a plate shaker (250 RPM) incubating at 37 C for 8 h, followed by dilutions/plating to estimate bacterial cytotoxicity. The remainder of the sample was spun down by centrifugation and used to estimate hemolysis (Fig. 3e).

### Statistics and reproducibility

Statistical analysis was performed using statistical packages included in GraphPad Prism 6. To assess the significance of increase in signal due to protease cleavage, we used a two-way ANOVA (without repeated measures) followed by Sidak’s multiple comparisons test. To assess the accuracy of assigning the binary value 0 or 1 to the digits p_0_ and p_1_ as seen in Fig. 2e, one-way unpaired t-tests were performed between the condition with GzmB and the negative control (no GzmB) for each respective output (i.e., p_0_ or p_1_) (Fig. 2e). A one-way ANOVA followed by Dunnett’s multiple comparisons test was used to compare experimental means to cells only control in Fig. 3d. Experiments in Figs. 1b, c, 2b, c, 3b, 4, and 5, were reproduced three times total with similar results. Experiments in Figs. 2e, 3c–e, and 7b were reproduced two times total with similar results. Experiments in Figs. 2d and 7a were performed once.

### Reporting summary

Further information on research design is available in the Nature Research Reporting Summary linked to this article.

## Supplementary information

Supplementary Information

Reporting Summary

## Data Availability

The authors declare that the data supporting the findings of this study are available within the paper and its supplementary information files. Any other relevant data are available upon reasonable request. Source data are provided with this paper.

## Source data

Source Data
