# Supplementary material for: Protease circuits for processing biological information
Source: Nat Commun. 2020 Oct 6;11:5021. doi: 10.1038/s41467-020-18840-8 (PMC7538567; doi:10.1038/s41467-020-18840-8)
Supplement: Supplementary file 1 — Supplementary Information [file 41467_2020_18840_MOESM1_ESM.pdf]

Supplementary Materials:

Supplementary Figs. 1 to 8
Supplementary Tables 1 to 2

Supplementary Figure 1

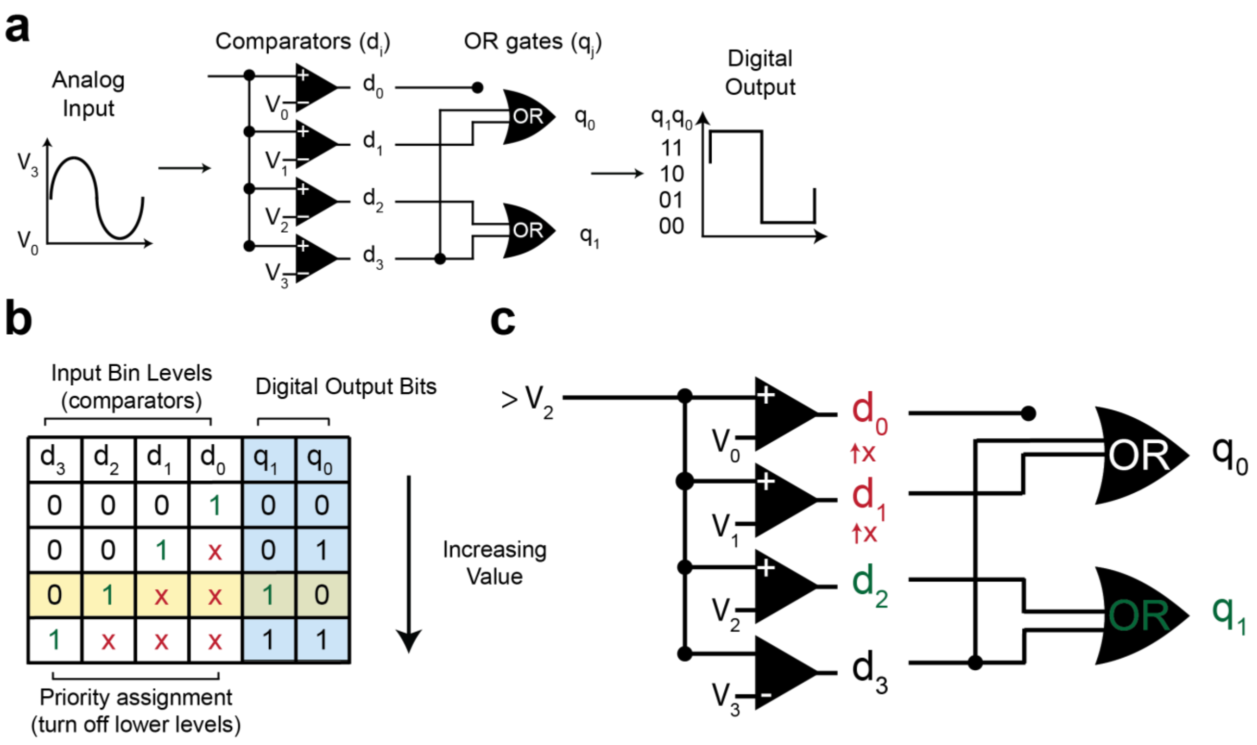

Supplementary Figure 1. Inputs and outputs of a 4-2 bit ADC. (a) Circuit diagram of a flash ADC. **(b)** Truth table. Inputs activate continuous subsets of the biocomparators (d_0 to d_3). An input which activates a biocomparator produces a value of 1. To give this input (d_n) priority, all biocomparators below (d_0-d_{n-1}) are turned off, signified by a value of x. Digital, two-valued output bits are colored blue and correspond to the 2-bit output of the ADC. **(c)** Logic circuit diagram for one example input/output case through a 4-2 bit ADC. As an example, input signal > V_2 turns on d_0—d_2, but priority is given to d_2, which only turns on bit q_1, producing the output 10.

Supplementary Figure 2

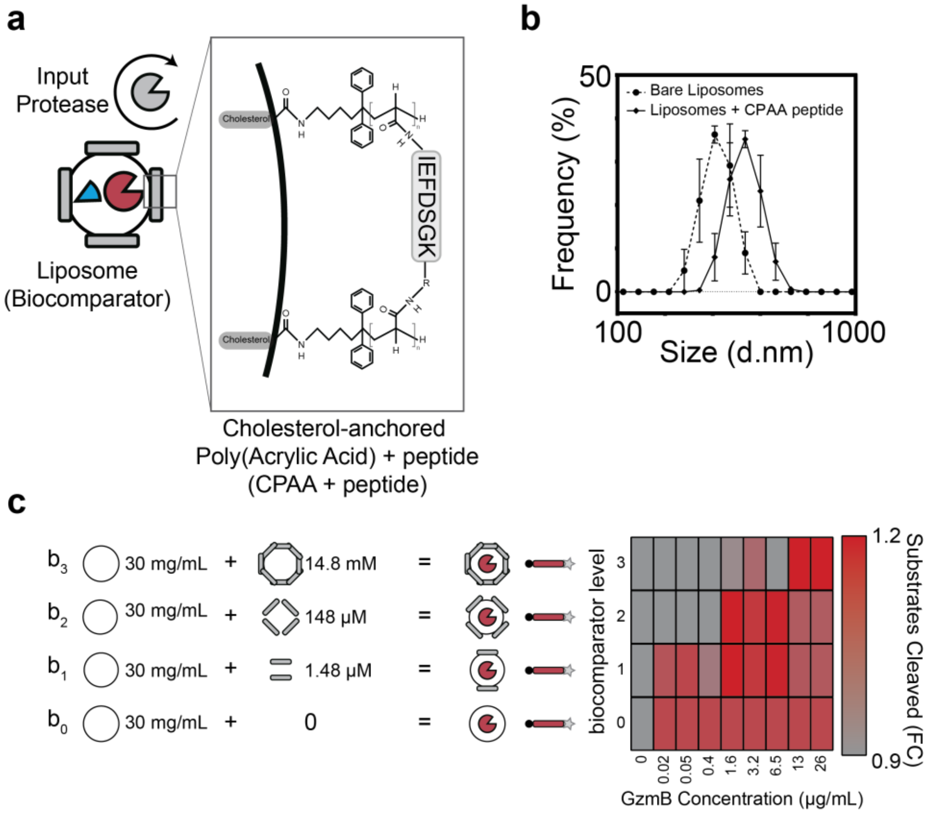

Supplementary Figure 2. Peptide caged liposome synthesis and characterization. (a) Graphic of cholesterol-anchored poly(acrylic acid) (CPAA) embedded in liposome membrane, crosslinked by amine terminated peptides. Carboxylic acid side groups on poly(acrylic acid) are activated by EDC*MeI. N-terminal amine and C-terminal amine (from lysine side chain) act as primary amines to react with activated CPAA side chains. **(b)** DLS size measurement of liposomes before and after peptide cage construction. Increase in average hydrodynamic radius from 264.0 nm (bare liposomes) to 344.3 nm (Liposomes + CPAA peptide). Data is presented as mean +/- s.e.m. (n = 3 biologically independent samples). **(c)** Heat map showing concentration of GzmB required to unlock each level. Signal is measured via released protease cutting substrate, normalized to the negative control (0 ug/mL signal protease). RFU stands for "Relative Fluorescence Unit" and is plotted as fold change (FC) from initial fluorescence at time = 0.

Supplementary Figure 3

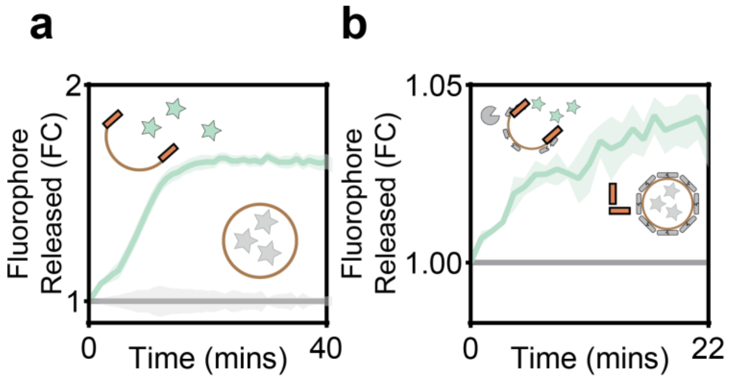

Supplementary Figure 3. Lipase acts as a Buffer gate. (a) Phospholipase C-triggered release of FITC contained in liposomes. Negative control contains FITC-loaded liposomes only and no lipase. Line shading represents standard deviation and line represents mean (n = 3 biologically independent samples). **(b)** Phospholipase-C and signal protease GzmB triggered release of FITC from FITC-loaded, peptide-caged liposomes. Negative control contains lipase plus FITC-loaded, peptide-caged liposomes, but no signal protease GzmB. Line shading represents standard deviation and line represents mean (n = 3 biologically independent samples for green line; n = 2 biologically independent samples for grey line). Released fluorophore (i.e., FITC) is quantified with units of RFU, which stands for "Relative Fluorescence Unit", and is plotted as fold change (FC) from initial fluorescence at time = 0.

Supplementary Figure 4

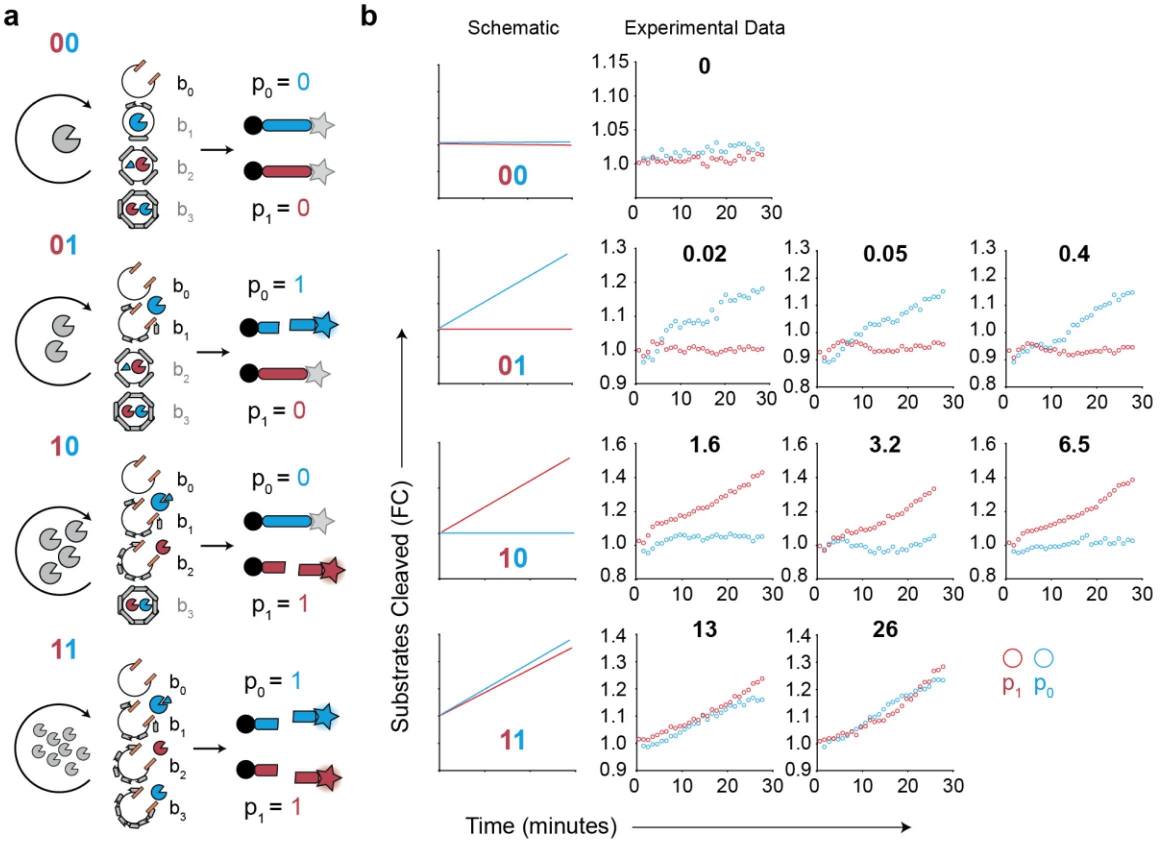

Supplementary Figure 4. Measuring all four possible outputs from biological analog-to-digital converter (ADC). (**a**) Schematic of all four possible signal conversions in the biological ADC. The signal protease (grey, GzmB), cleaves the peptide cage surrounding biocomparators. Higher activity levels of GzmB result in more biocomparator levels being unlocked (b_0 to b_3). Lipase (orange rectangle) is co-incubated with the bioADC such that all exposed biocomparators are fully opened via degradation of liposome by phospholipase C. Released proteases and inhibitors interact to produce a two-valued, digital signal (i.e., 00, 01, 10, 11). The resulting proteases interact with OR gates to produce "high", or 1, values for the correct two-valued outputs. (**b**) Kinetic fluorescence data from bioADC circuit. Blue dots represent signal from p_0 substrate, and red dots represent signal from p_1 substrate. Bold numbers represent the input concentration of GzmB in ug/mL. Amount of substrates cleaved is quantified with units of RFU, which stands for "Relative Fluorescence Unit", and is plotted as fold change (FC) from initial fluorescence at time = 0.

Supplementary Figure 5

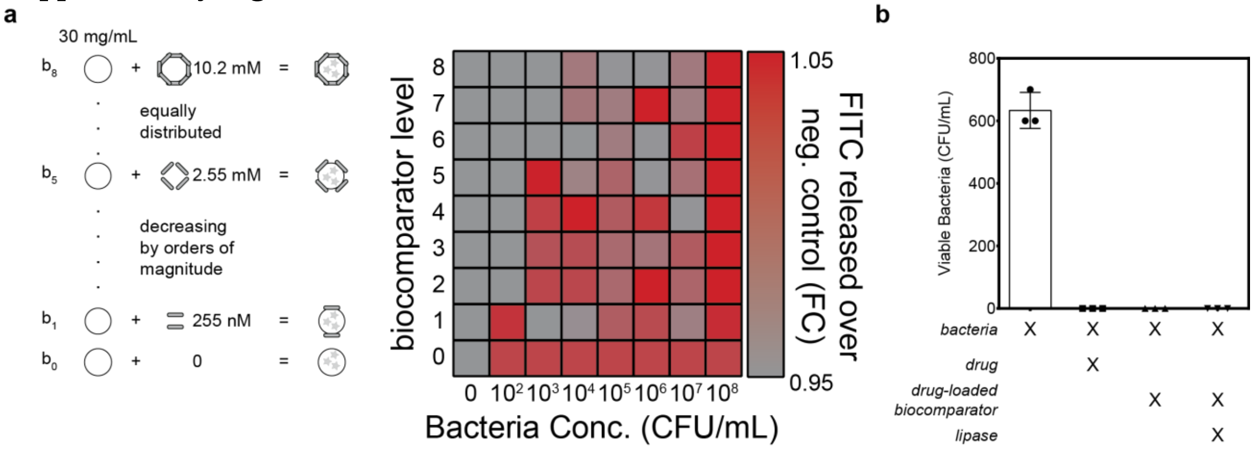

Supplementary Figure 5. Biocomparator activation levels and examining the behavior drug-loaded biocomparators. (a) Unlocking peptide caged liposomes (i.e., biocomparators) with increasing peptide crosslinking densities (levels 0 – 8). Eight levels of increasing peptide cage crosslinking densities were used to determine the number of bacteria required to unlock each level. Increased concentration of input protease (OmpT, from *E. coli*) leads to more unlocked levels. **(b)** Bacterial cytotoxicity measurement of drug-loaded biocomparators. Conditions are moving from left to right: Bacteria only control, bacteria plus free drug, bacteria plus drug-loaded liposome without lipase, and bacteria plus drug-loaded liposome with lipase. Samples were incubated with bacteria at 37C for eight hours and plated. CFU were quantified to estimate bacteria viability. Data is plotted as mean +/- standard deviation (n = 3 biologically independent samples). RFU stands for "Relative Fluorescence Unit" and is plotted as fold change (FC) from initial fluorescence at time = 0.

Supplementary Figure 6

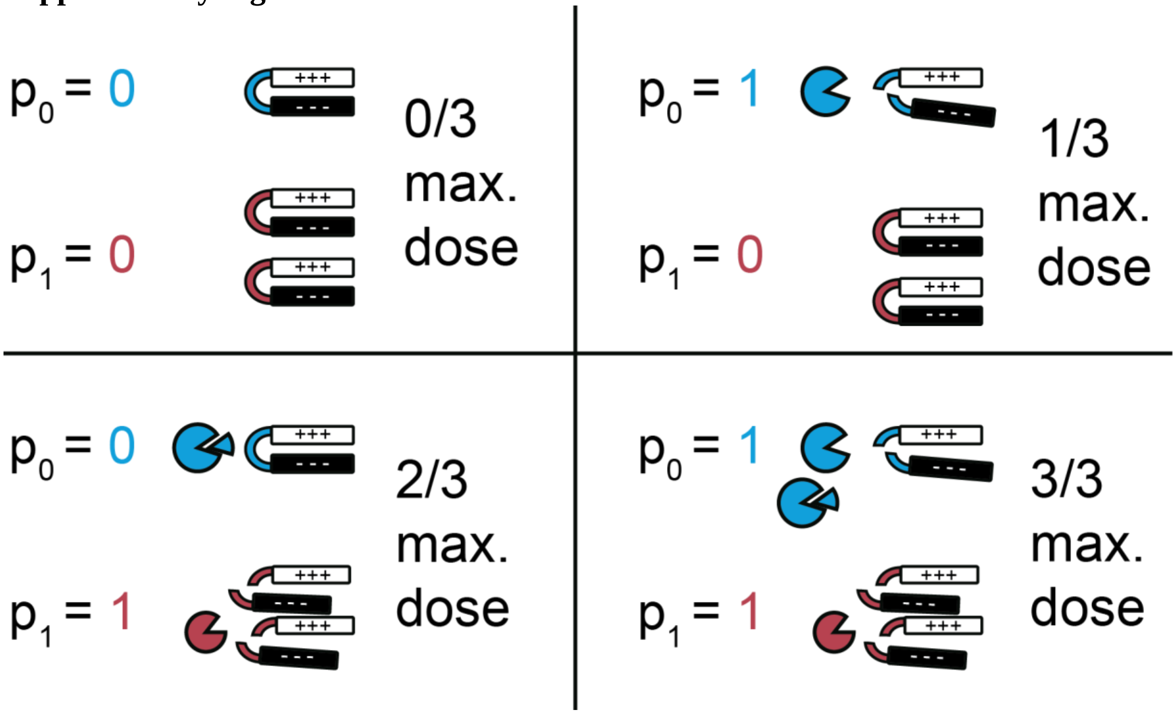

Supplementary Figure 6. All possible output drug doses from biological ADC. The OR gate linked to p_0 (blue substrate) outputs 1/3 of the available AMP dose, whereas the OR gate linked to p_1 (red substrate) outputs 2/3 of the available AMP dose. This translates each digital output to a drug dose increasing by units of 1/3 the total dose. For example, when only p_0 is active (i.e., $p_0 = 1$, $p_1 = 0$), 1/3 of the maximum drug dose can be unlocked, whereas if only p_1 is active (i.e., $p_0 = 0$, $p_1 = 1$), then 2/3 of the maximum drug dose can be unlocked.

Supplementary Figure 7

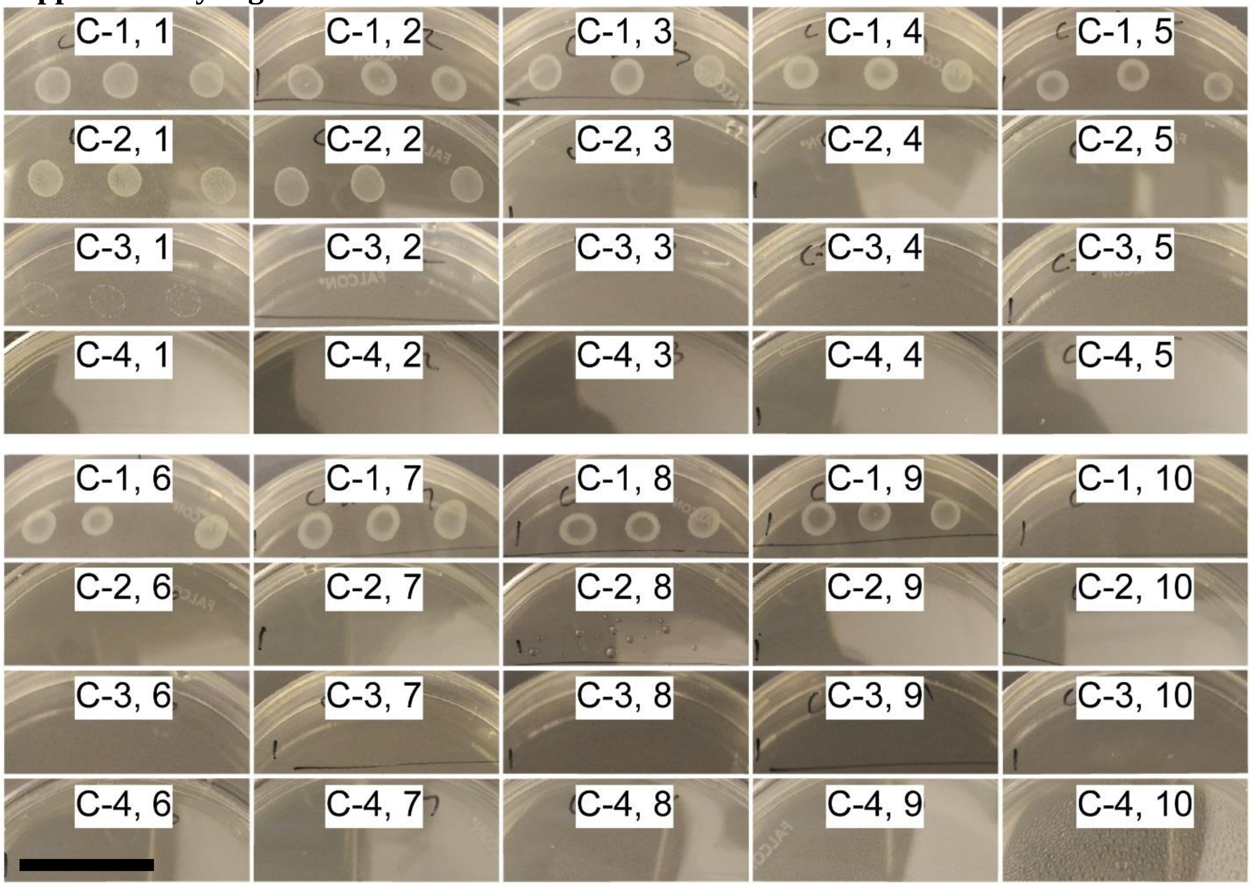

Supplementary Figure 7. Photos of bacterial plates post-incubation with bioprograms. Photos of bacterial plates used for quantification of cytotoxicity in Fig 3e. C-1 indicates the single, empty comparator bioprogram control, which represents 100% cell viability, or 0% cytotoxicity. No colony growth represents 0% cell viability, or 100% cytotoxicity. Plates are labeled C-*n X*, where *n* corresponds to the number of biocomparators p resent in the program combined with infected blood and *X* corresponds to the concentration of bacteria present. (10 = 10^0 CFU/mL, 9 = 10^1 CFU/mL, 8 = 10^2 CFU/mL, etc.). Scale bar (bottom left) = 30 mm.

Supplemental Figure 8

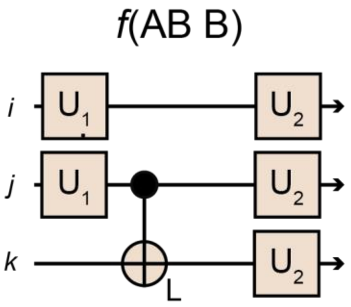

Example Ideal Oracle problem - *f*(AB B)

Signal	Initial		U_1 Operation		L Operation		U_2 Operation	
	f(A)	*f*(B)	*f*(A)	*f*(B)	*f*(A)	*f*(B)	*f*(A)	*f*(B)
i	100	0	50	50	50	50	100	0
j	100	0	50	50	0	100	0	100
k	100	0	100	0	0	100	0	100

Supplementary Figure 8. Example of ideal solution for oracle problem - *f*(AB B). Table depicting fractional cleavage outputs of the three signals (i.e., *i*, *j*, and *k*) that constitute the joint-state (i.e., joint-state = [*ij k*]) after each operation. Each column represents the fractional cleavage values for the given signal after the operation for the column is applied. Correct final joint-state is *f*(*ij k*) = *f*(AB B), where *i* = A, *k* = B, and *k* = B. For example, the final fractional cleavage scores for each signal, respectively, are: signal *i*, *f*(A) = 100%, signal *j*, *f*(B) = 100%, and signal *k*, *f*(B) = 100%.

Supplementary Table 1

**Name**	**Abbreviation**	**Substrate(s)***	**Inhibitor****
Granzyme B Protease	GzmB	**5FAM**-aIEFDSGK-**CPQ2**-kkc	NA
West Nile Virus Protease	WNVp	**5TAMRA**-RTKR-**QXL570**	undeca-D-Arg-**NH_2**
Tobacco Etch Virus Protease	TEVp	**5FAM**-ENLYFQG-**QXL520**	NA
Outer Membrane Protein T	OmpT	**DABCYL**-RRSRRVK-**5FAM**	NA

*Lower-case letters symbolize d-amino acids
*Bold strings are non-amino acid groups, as labeled below
***5FAM** = 5 – Carboxyfluorescein
***DABCYL** = 4-((4-(dimethylamino)phenyl)azo)benzoic acid
***QXL570**, **QXL520** = Proprietary quencher from Anaspec, Inc (Fremont, CA)
***CPQ2** = Proprietary quencher from CPC, Scientific (Sunnyvale, CA)
***5TAMRA** = 5-Carboxytetramethylrhodamine
****** undeca-D-Arg-NH_2 = 11 D-arginine motifs (i.e., rrrrrrrrrrr); **NH_2** = c-terminal amidation

Supplementary Table 2

**Name**	**Peptide Sequence***
Polyarginine cationic AMP	RRRRRRRR
Protease-activatable, Drug-locked peptide, p_0	EEEEEEEEEEERKTRRRRRRRRR
Protease-activatable, Drug-locked peptide, p_1	EEEEEEEEENLYFQGRRRRRRRRR

*****All motifs are l-amino acids, without any N- or C- terminal modifications
